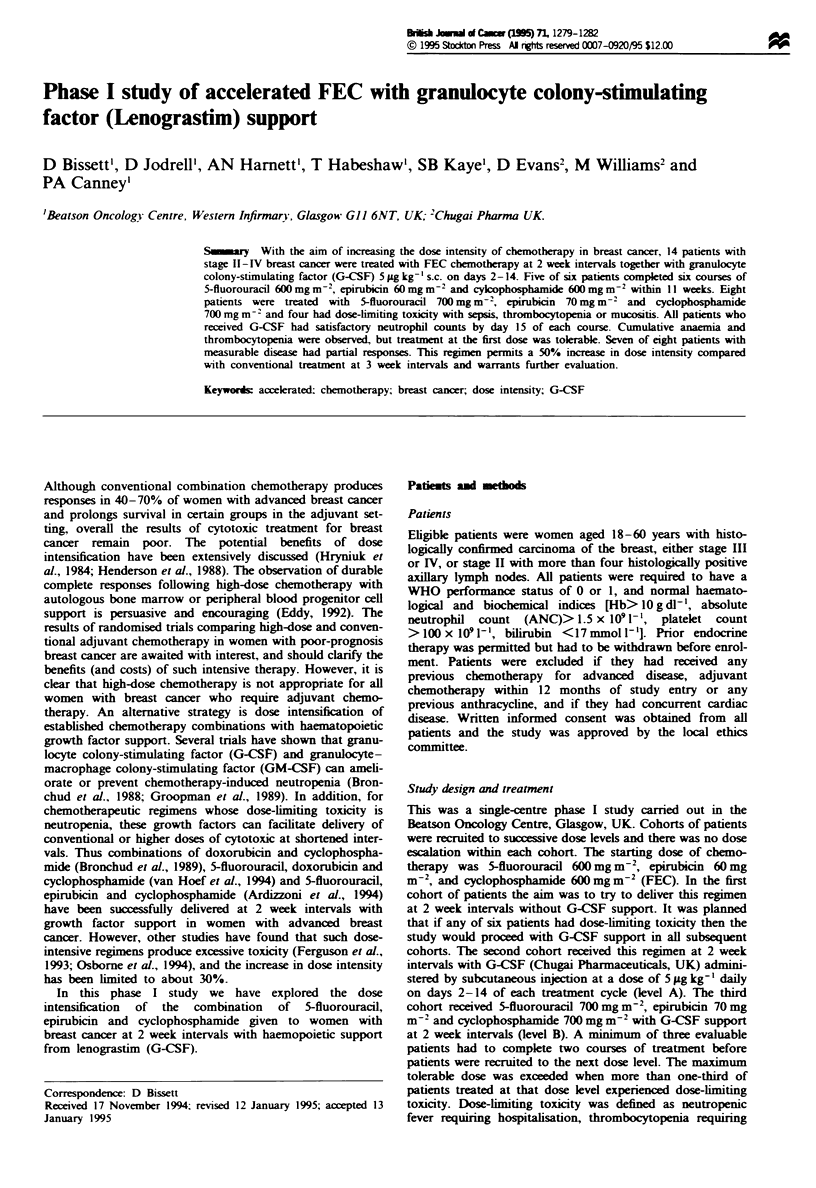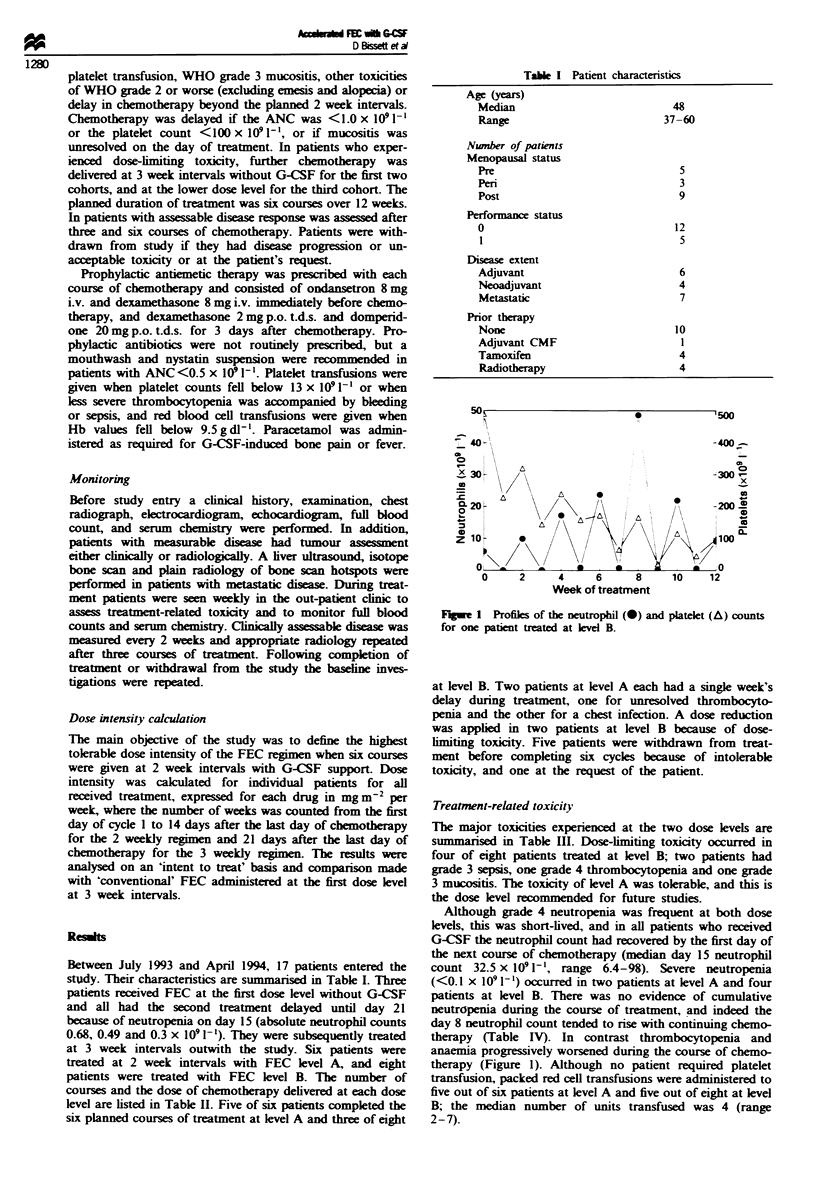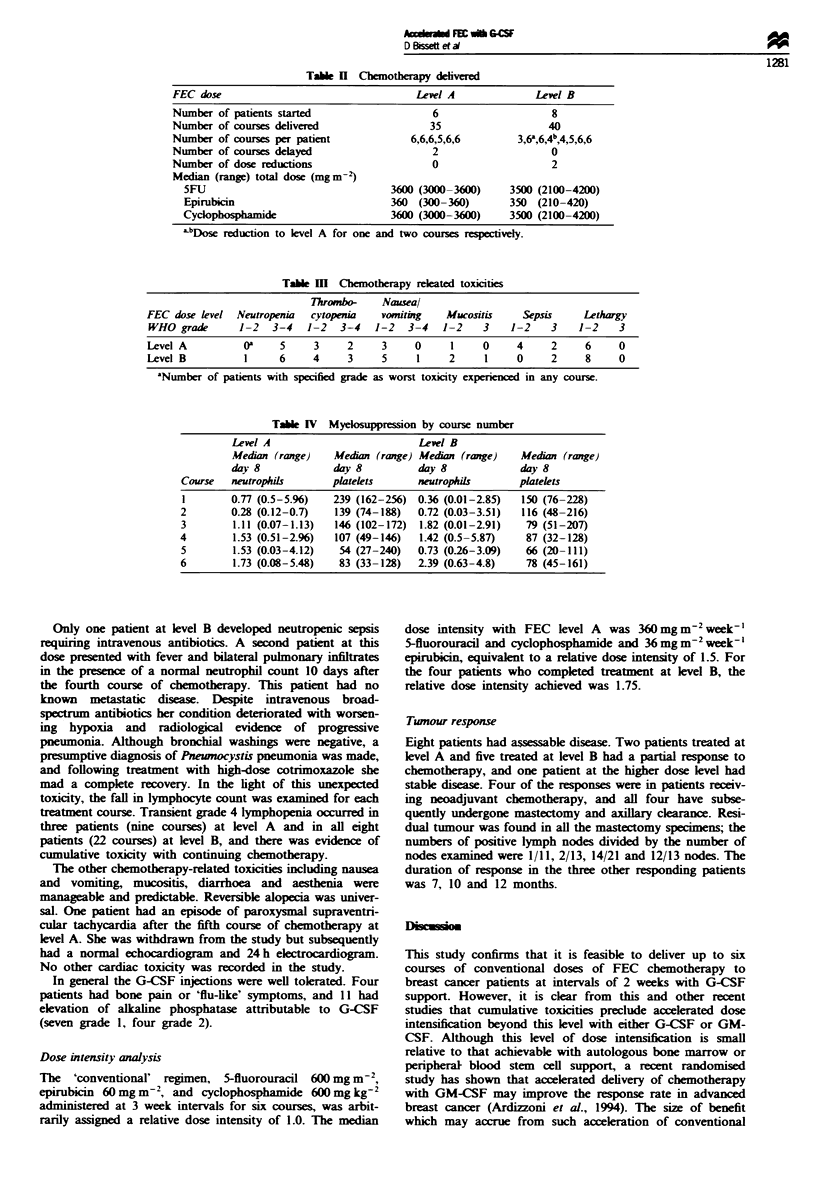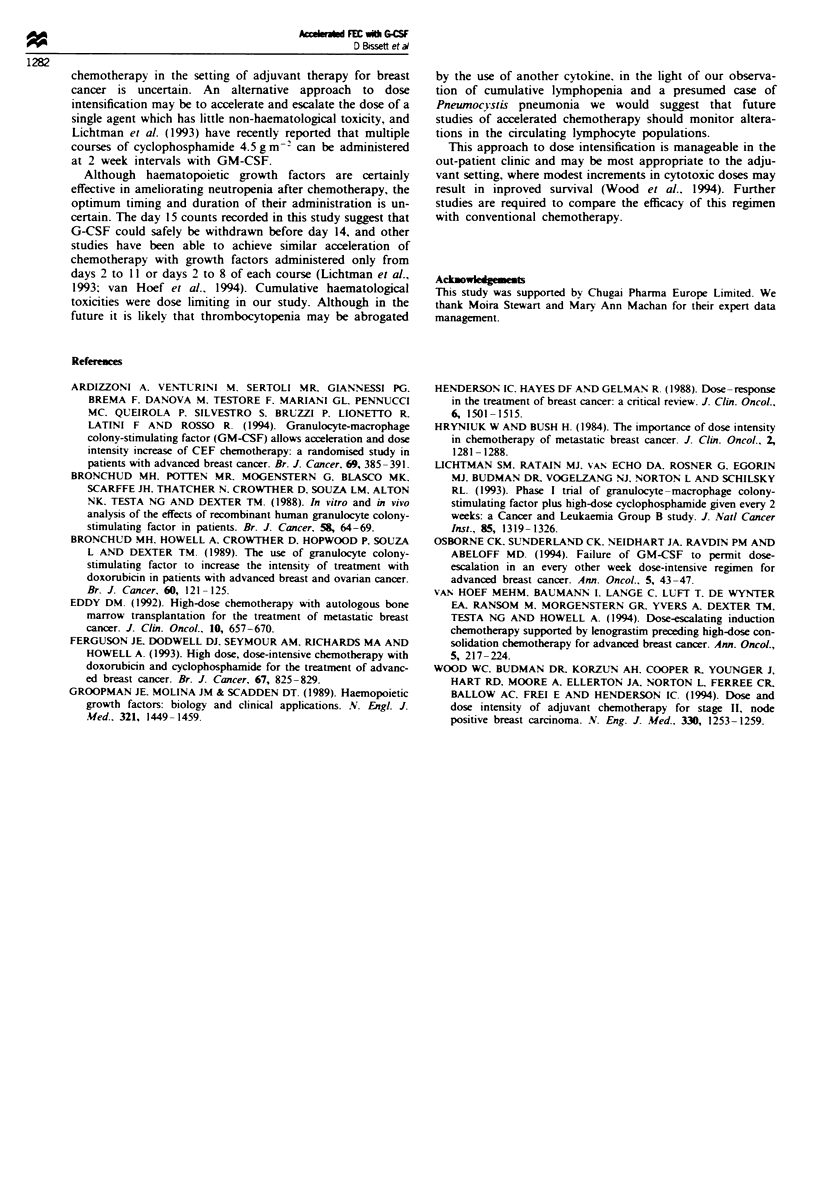# Phase I study of accelerated FEC with granulocyte colony-stimulating factor (Lenograstim) support.

**DOI:** 10.1038/bjc.1995.247

**Published:** 1995-06

**Authors:** D. Bissett, D. Jodrell, A. N. Harnett, T. Habeshaw, S. B. Kaye, D. Evans, M. Williams, P. A. Canney

**Affiliations:** Beatson Oncology Centre, Western Infirmary, Glasgow, UK.

## Abstract

With the aim of increasing the dose intensity of chemotherapy in breast cancer, 14 patients with stage II-IV breast cancer were treated with FEC chemotherapy at 2 week intervals together with granulocyte colony-stimulating factor (G-CSF) 5 micrograms kg-1 s.c. on days 2-14. Five of six patients completed six courses of 5-fluorouracil 600 mg m-2, epirubicin 60 mg m-2 and cylcophosphamide 600 mg m-2 within 11 weeks. Eight patients were treated with 5-fluorouracil 700 mg m-2, epirubicin 70 mg m-2 and cyclophosphamide 700 mg m-2 and four had dose-limiting toxicity with sepsis, thrombocytopenia or mucositis. All patients who received G-CSF had satisfactory neutrophil counts by day 15 of each course. Cumulative anaemia and thrombocytopenia were observed, but treatment at the first dose was tolerable. Seven of eight patients with measurable disease had partial responses. This regimen permits a 50% increase in dose intensity compared with conventional treatment at 3 week intervals and warrants further evaluation.


© 1995 Stockton Press All rights reserved 0007-0920/95 $12.00

### Phase I study of accelerated FEC with granulocyte colony-stimulating factor (Lenograstim) support

D Bissett[1], D Jodrell[1], AN Harnett[1], T Habeshaw[1], SB Kaye[1], D Evans[2], M Williams[2] and PA Canney[1]

[1]*Beatson Oncology Centre, Western Infirmary, Glasgow G11 6NT, UK;* [2]*Chugai Pharma UK.*

**Summary** With the aim of increasing the dose intensity of chemotherapy in breast cancer, 14 patients with stage II-IV breast cancer were treated with FEC chemotherapy at 2 week intervals together with granulocyte colony-stimulating factor (G-CSF) 5 $\mu$g $kg^{-1}$ s.c. on days 2-14. Five of six patients completed six courses of 5-fluorouracil 600 mg $m^{-2}$, epirubicin 60 mg $m^{-2}$ and cylcophosphamide 600 mg $m^{-2}$ within 11 weeks. Eight patients were treated with 5-fluorouracil 700 mg $m^{-2}$, epirubicin 70 mg $m^{-2}$ and cyclophosphamide 700 mg $m^{-2}$ and four had dose-limiting toxicity with sepsis, thrombocytopenia or mucositis. All patients who received G-CSF had satisfactory neutrophil counts by day 15 of each course. Cumulative anaemia and thrombocytopenia were observed, but treatment at the first dose was tolerable. Seven of eight patients with measurable disease had partial responses. This regimen permits a 50% increase in dose intensity compared with conventional treatment at 3 week intervals and warrants further evaluation.

**Keywords:** accelerated; chemotherapy; breast cancer; dose intensity; G-CSF

Although conventional combination chemotherapy produces responses in 40-70% of women with advanced breast cancer and prolongs survival in certain groups in the adjuvant setting, overall the results of cytotoxic treatment for breast cancer remain poor. The potential benefits of dose intensification have been extensively discussed (Hryniuk *et al.*, 1984; Henderson *et al.*, 1988). The observation of durable complete responses following high-dose chemotherapy with autologous bone marrow or peripheral blood progenitor cell support is persuasive and encouraging (Eddy, 1992). The results of randomised trials comparing high-dose and conventional adjuvant chemotherapy in women with poor-prognosis breast cancer are awaited with interest, and should clarify the benefits (and costs) of such intensive therapy. However, it is clear that high-dose chemotherapy is not appropriate for all women with breast cancer who require adjuvant chemotherapy. An alternative strategy is dose intensification of established chemotherapy combinations with haematopoietic growth factor support. Several trials have shown that granulocyte colony-stimulating factor (G-CSF) and granulocyte-macrophage colony-stimulating factor (GM-CSF) can ameliorate or prevent chemotherapy-induced neutropenia (Bronchud *et al.*, 1988; Groopman *et al.*, 1989). In addition, for chemotherapeutic regimens whose dose-limiting toxicity is neutropenia, these growth factors can facilitate delivery of conventional or higher doses of cytotoxic at shortened intervals. Thus combinations of doxorubicin and cyclophosphamide (Bronchud *et al.*, 1989), 5-fluorouracil, doxorubicin and cyclophosphamide (van Hoef *et al.*, 1994) and 5-fluorouracil, epirubicin and cyclophosphamide (Ardizzoni *et al.*, 1994) have been successfully delivered at 2 week intervals with growth factor support in women with advanced breast cancer. However, other studies have found that such dose-intensive regimens produce excessive toxicity (Ferguson *et al.*, 1993; Osborne *et al.*, 1994), and the increase in dose intensity has been limited to about 30%.

In this phase I study we have explored the dose intensification of the combination of 5-fluorouracil, epirubicin and cyclophosphamide given to women with breast cancer at 2 week intervals with haemopoietic support from lenograstim (G-CSF).

Correspondence: D Bissett
Received 17 November 1994; revised 12 January 1995; accepted 13 January 1995

### Patients and methods

### *Patients*

Eligible patients were women aged 18-60 years with histologically confirmed carcinoma of the breast, either stage III or IV, or stage II with more than four histologically positive axillary lymph nodes. All patients were required to have a WHO performance status of 0 or 1, and normal haematological and biochemical indices [Hb > 10 g $dl^{-1}$, absolute neutrophil count (ANC) > $1.5 \times 10^9 l^{-1}$, platelet count > $100 \times 10^9 l^{-1}$, bilirubin < 17 mmol $l^{-1}$]. Prior endocrine therapy was permitted but had to be withdrawn before enrolment. Patients were excluded if they had received any previous chemotherapy for advanced disease, adjuvant chemotherapy within 12 months of study entry or any previous anthracycline, and if they had concurrent cardiac disease. Written informed consent was obtained from all patients and the study was approved by the local ethics committee.

### *Study design and treatment*

This was a single-centre phase I study carried out in the Beatson Oncology Centre, Glasgow, UK. Cohorts of patients were recruited to successive dose levels and there was no dose escalation within each cohort. The starting dose of chemotherapy was 5-fluorouracil 600 mg $m^{-2}$, epirubicin 60 mg $m^{-2}$, and cyclophosphamide 600 mg $m^{-2}$ (FEC). In the first cohort of patients the aim was to try to deliver this regimen at 2 week intervals without G-CSF support. It was planned that if any of six patients had dose-limiting toxicity then the study would proceed with G-CSF support in all subsequent cohorts. The second cohort received this regimen at 2 week intervals with G-CSF (Chugai Pharmaceuticals, UK) administered by subcutaneous injection at a dose of 5 $\mu$g $kg^{-1}$ daily on days 2-14 of each treatment cycle (level A). The third cohort received 5-fluorouracil 700 mg $m^{-2}$, epirubicin 70 mg $m^{-2}$ and cyclophosphamide 700 mg $m^{-2}$ with G-CSF support at 2 week intervals (level B). A minimum of three evaluable patients had to complete two courses of treatment before patients were recruited to the next dose level. The maximum tolerable dose was exceeded when more than one-third of patients treated at that dose level experienced dose-limiting toxicity. Dose-limiting toxicity was defined as neutropenic fever requiring hospitalisation, thrombocytopenia requiring platelet transfusion, WHO grade 3 mucositis, other toxicities of WHO grade 2 or worse (excluding emesis and alopecia) or delay in chemotherapy beyond the planned 2 week intervals. Chemotherapy was delayed if the ANC was $<1.0 \times 10^9 l^{-1}$ or the platelet count $<100 \times 10^9 l^{-1}$, or if mucositis was unresolved on the day of treatment. In patients who experienced dose-limiting toxicity, further chemotherapy was delivered at 3 week intervals without G-CSF for the first two cohorts, and at the lower dose level for the third cohort. The planned duration of treatment was six courses over 12 weeks. In patients with assessable disease response was assessed after three and six courses of chemotherapy. Patients were withdrawn from study if they had disease progression or unacceptable toxicity or at the patient's request.

Prophylactic antiemetic therapy was prescribed with each course of chemotherapy and consisted of ondansetron 8 mg i.v. and dexamethasone 8 mg i.v. immediately before chemotherapy, and dexamethasone 2 mg p.o. t.d.s. and domperidone 20 mg p.o. t.d.s. for 3 days after chemotherapy. Prophylactic antibiotics were not routinely prescribed, but a mouthwash and nystatin suspension were recommended in patients with ANC $<0.5 \times 10^9 l^{-1}$. Platelet transfusions were given when platelet counts fell below $13 \times 10^9 l^{-1}$ or when less severe thrombocytopenia was accompanied by bleeding or sepsis, and red blood cell transfusions were given when Hb values fell below $9.5 g dl^{-1}$. Paracetamol was administered as required for G-CSF-induced bone pain or fever.

### *Monitoring*

Before study entry a clinical history, examination, chest radiograph, electrocardiogram, echocardiogram, full blood count, and serum chemistry were performed. In addition, patients with measurable disease had tumour assessment either clinically or radiologically. A liver ultrasound, isotope bone scan and plain radiology of bone scan hotspots were performed in patients with metastatic disease. During treatment patients were seen weekly in the out-patient clinic to assess treatment-related toxicity and to monitor full blood counts and serum chemistry. Clinically assessable disease was measured every 2 weeks and appropriate radiology repeated after three courses of treatment. Following completion of treatment or withdrawal from the study the baseline investigations were repeated.

### *Dose intensity calculation*

The main objective of the study was to define the highest tolerable dose intensity of the FEC regimen when six courses were given at 2 week intervals with G-CSF support. Dose intensity was calculated for individual patients for all received treatment, expressed for each drug in $mg m^{-2}$ per week, where the number of weeks was counted from the first day of cycle 1 to 14 days after the last day of chemotherapy for the 2 weekly regimen and 21 days after the last day of chemotherapy for the 3 weekly regimen. The results were analysed on an 'intent to treat' basis and comparison made with 'conventional' FEC administered at the first dose level at 3 week intervals.

### Results

Between July 1993 and April 1994, 17 patients entered the study. Their characteristics are summarised in Table I. Three patients received FEC at the first dose level without G-CSF and all had the second treatment delayed until day 21 because of neutropenia on day 15 (absolute neutrophil counts 0.68, 0.49 and $0.3 \times 10^9 l^{-1}$). They were subsequently treated at 3 week intervals outwith the study. Six patients were treated at 2 week intervals with FEC level A, and eight patients were treated with FEC level B. The number of courses and the dose of chemotherapy delivered at each dose level are listed in Table II. Five of six patients completed the six planned courses of treatment at level A and three of eight

**Table I** Patient characteristics

| | |
|---|---|
| Age (years) | |
| Median | 48 |
| Range | 37–60 |
| *Number of patients* | |
| Menopausal status | |
| Pre | 5 |
| Peri | 3 |
| Post | 9 |
| Performance status | |
| 0 | 12 |
| 1 | 5 |
| Disease extent | |
| Adjuvant | 6 |
| Neoadjuvant | 4 |
| Metastatic | 7 |
| Prior therapy | |
| None | 10 |
| Adjuvant CMF | 1 |
| Tamoxifen | 4 |
| Radiotherapy | 4 |

**Figure 1** Profiles of the neutrophil (●) and platelet (Δ) counts for one patient treated at level B.

at level B. Two patients at level A each had a single week's delay during treatment, one for unresolved thrombocytopenia and the other for a chest infection. A dose reduction was applied in two patients at level B because of dose-limiting toxicity. Five patients were withdrawn from treatment before completing six cycles because of intolerable toxicity, and one at the request of the patient.

### *Treatment-related toxicity*

The major toxicities experienced at the two dose levels are summarised in Table III. Dose-limiting toxicity occurred in four of eight patients treated at level B; two patients had grade 3 sepsis, one grade 4 thrombocytopenia and one grade 3 mucositis. The toxicity of level A was tolerable, and this is the dose level recommended for future studies.

Although grade 4 neutropenia was frequent at both dose levels, this was short-lived, and in all patients who received G-CSF the neutrophil count had recovered by the first day of the next course of chemotherapy (median day 15 neutrophil count $32.5 \times 10^9 l^{-1}$, range 6.4–98). Severe neutropenia ($<0.1 \times 10^9 l^{-1}$) occurred in two patients at level A and four patients at level B. There was no evidence of cumulative neutropenia during the course of treatment, and indeed the day 8 neutrophil count tended to rise with continuing chemotherapy (Table IV). In contrast thrombocytopenia and anaemia progressively worsened during the course of chemotherapy (Figure 1). Although no patient required platelet transfusion, packed red cell transfusions were administered to five out of six patients at level A and five out of eight at level B; the median number of units transfused was 4 (range 2–7).

**Table II** Chemotherapy delivered

| *FEC dose* | *Level A* | *Level B* |
|---|---|---|
| Number of patients started | 6 | 8 |
| Number of courses delivered | 35 | 40 |
| Number of courses per patient | 6,6,6,5,6,6 | 3,6[a],6,4[b],4,5,6,6 |
| Number of courses delayed | 2 | 0 |
| Number of dose reductions | 0 | 2 |
| Median (range) total dose (mg $m^{-2}$) | | |
| 5FU | 3600 (3000-3600) | 3500 (2100-4200) |
| Epirubicin | 360 (300-360) | 350 (210-420) |
| Cyclophosphamide | 3600 (3000-3600) | 3500 (2100-4200) |

[a,b]Dose reduction to level A for one and two courses respectively.

**Table III** Chemotherapy releated toxicities

| *FEC dose level* | *Neutropenia* | | *Thrombo-cytopenia* | | *Nausea/ vomiting* | | *Mucositis* | | *Sepsis* | | *Lethargy* | |
|---|---|---|---|---|---|---|---|---|---|---|---|---|
| *WHO grade* | *1-2* | *3-4* | *1-2* | *3-4* | *1-2* | *3-4* | *1-2* | *3* | *1-2* | *3* | *1-2* | *3* |
| Level A | 0[a] | 5 | 3 | 2 | 3 | 0 | 1 | 0 | 4 | 2 | 6 | 0 |
| Level B | 1 | 6 | 4 | 3 | 5 | 1 | 2 | 1 | 0 | 2 | 8 | 0 |

[a]Number of patients with specified grade as worst toxicity experienced in any course.

**Table IV** Myelosuppression by course number

| *Course* | *Level A* *Median (range) day 8 neutrophils* | *Median (range) day 8 platelets* | *Level B* *Median (range) day 8 neutrophils* | *Median (range) day 8 platelets* |
|---|---|---|---|---|
| 1 | 0.77 (0.5-5.96) | 239 (162-256) | 0.36 (0.01-2.85) | 150 (76-228) |
| 2 | 0.28 (0.12-0.7) | 139 (74-188) | 0.72 (0.03-3.51) | 116 (48-216) |
| 3 | 1.11 (0.07-1.13) | 146 (102-172) | 1.82 (0.01-2.91) | 79 (51-207) |
| 4 | 1.53 (0.51-2.96) | 107 (49-146) | 1.42 (0.5-5.87) | 87 (32-128) |
| 5 | 1.53 (0.03-4.12) | 54 (27-240) | 0.73 (0.26-3.09) | 66 (20-111) |
| 6 | 1.73 (0.08-5.48) | 83 (33-128) | 2.39 (0.63-4.8) | 78 (45-161) |

Only one patient at level B developed neutropenic sepsis requiring intravenous antibiotics. A second patient at this dose presented with fever and bilateral pulmonary infiltrates in the presence of a normal neutrophil count 10 days after the fourth course of chemotherapy. This patient had no known metastatic disease. Despite intravenous broad-spectrum antibiotics her condition deteriorated with worsening hypoxia and radiological evidence of progressive pneumonia. Although bronchial washings were negative, a presumptive diagnosis of *Pneumocystis* pneumonia was made, and following treatment with high-dose cotrimoxazole she mad a complete recovery. In the light of this unexpected toxicity, the fall in lymphocyte count was examined for each treatment course. Transient grade 4 lymphopenia occurred in three patients (nine courses) at level A and in all eight patients (22 courses) at level B, and there was evidence of cumulative toxicity with continuing chemotherapy.

The other chemotherapy-related toxicities including nausea and vomiting, mucositis, diarrhoea and aesthenia were manageable and predictable. Reversible alopecia was universal. One patient had an episode of paroxysmal supraventricular tachycardia after the fifth course of chemotherapy at level A. She was withdrawn from the study but subsequently had a normal echocardiogram and 24 h electrocardiogram. No other cardiac toxicity was recorded in the study.

In general the G-CSF injections were well tolerated. Four patients had bone pain or 'flu-like' symptoms, and 11 had elevation of alkaline phosphatase attributable to G-CSF (seven grade 1, four grade 2).

### *Dose intensity analysis*

The 'conventional' regimen, 5-fluorouracil 600 mg $m^{-2}$, epirubicin 60 mg $m^{-2}$, and cyclophosphamide 600 mg $kg^{-2}$ administered at 3 week intervals for six courses, was arbitrarily assigned a relative dose intensity of 1.0. The median dose intensity with FEC level A was 360 mg $m^{-2}$ $week^{-1}$ 5-fluorouracil and cyclophosphamide and 36 mg $m^{-2}$ $week^{-1}$ epirubicin, equivalent to a relative dose intensity of 1.5. For the four patients who completed treatment at level B, the relative dose intensity achieved was 1.75.

### *Tumour response*

Eight patients had assessable disease. Two patients treated at level A and five treated at level B had a partial response to chemotherapy, and one patient at the higher dose level had stable disease. Four of the responses were in patients receiving neoadjuvant chemotherapy, and all four have subsequently undergone mastectomy and axillary clearance. Residual tumour was found in all the mastectomy specimens; the numbers of positive lymph nodes divided by the number of nodes examined were 1/11, 2/13, 14/21 and 12/13 nodes. The duration of response in the three other responding patients was 7, 10 and 12 months.

### Discussion

This study confirms that it is feasible to deliver up to six courses of conventional doses of FEC chemotherapy to breast cancer patients at intervals of 2 weeks with G-CSF support. However, it is clear from this and other recent studies that cumulative toxicities preclude accelerated dose intensification beyond this level with either G-CSF or GM-CSF. Although this level of dose intensification is small relative to that achievable with autologous bone marrow or peripheral blood stem cell support, a recent randomised study has shown that accelerated delivery of chemotherapy with GM-CSF may improve the response rate in advanced breast cancer (Ardizzoni *et al.*, 1994). The size of benefit which may accrue from such acceleration of conventional chemotherapy in the setting of adjuvant therapy for breast cancer is uncertain. An alternative approach to dose intensification may be to accelerate and escalate the dose of a single agent which has little non-haematological toxicity, and Lichtman *et al.* (1993) have recently reported that multiple courses of cyclophosphamide 4.5 g $m^{-2}$ can be administered at 2 week intervals with GM-CSF.

Although haematopoietic growth factors are certainly effective in ameliorating neutropenia after chemotherapy, the optimum timing and duration of their administration is uncertain. The day 15 counts recorded in this study suggest that G-CSF could safely be withdrawn before day 14, and other studies have been able to achieve similar acceleration of chemotherapy with growth factors administered only from days 2 to 11 or days 2 to 8 of each course (Lichtman *et al.*, 1993; van Hoef *et al.*, 1994). Cumulative haematological toxicities were dose limiting in our study. Although in the future it is likely that thrombocytopenia may be abrogated by the use of another cytokine, in the light of our observation of cumulative lymphopenia and a presumed case of *Pneumocystis* pneumonia we would suggest that future studies of accelerated chemotherapy should monitor alterations in the circulating lymphocyte populations.

This approach to dose intensification is manageable in the out-patient clinic and may be most appropriate to the adjuvant setting, where modest increments in cytotoxic doses may result in inproved survival (Wood *et al.*, 1994). Further studies are required to compare the efficacy of this regimen with conventional chemotherapy.

**Acknowledgements**

This study was supported by Chugai Pharma Europe Limited. We thank Moira Stewart and Mary Ann Machan for their expert data management.

### References

ARDIZZONI A, VENTURINI M, SERTOLI MR, GIANNESSI PG, BREMA F, DANOVA M, TESTORE F, MARIANI GL, PENNUCCI MC, QUEIROLA P, SILVESTRO S, BRUZZI P, LIONETTO R, LATINI F AND ROSSO R. (1994). Granulocyte-macrophage colony-stimulating factor (GM-CSF) allows acceleration and dose intensity increase of CEF chemotherapy: a randomised study in patients with advanced breast cancer. *Br. J. Cancer*, **69**, 385-391.

BRONCHUD MH, POTTEN MR, MOGENSTERN G, BLASCO MK, SCARFFE JH, THATCHER N, CROWTHER D, SOUZA LM, ALTON NK, TESTA NG AND DEXTER TM. (1988). *In vitro* and *in vivo* analysis of the effects of recombinant human granulocyte colony-stimulating factor in patients. *Br. J. Cancer*, **58**, 64-69.

BRONCHUD MH, HOWELL A, CROWTHER D, HOPWOOD P, SOUZA L AND DEXTER TM. (1989). The use of granulocyte colony-stimulating factor to increase the intensity of treatment with doxorubicin in patients with advanced breast and ovarian cancer. *Br. J. Cancer*, **60**, 121-125.

EDDY DM. (1992). High-dose chemotherapy with autologous bone marrow transplantation for the treatment of metastatic breast cancer. *J. Clin. Oncol.*, **10**, 657-670.

FERGUSON JE, DODWELL DJ, SEYMOUR AM, RICHARDS MA AND HOWELL A. (1993). High dose, dose-intensive chemotherapy with doxorubicin and cyclophosphamide for the treatment of advanced breast cancer. *Br. J. Cancer*, **67**, 825-829.

GROOPMAN JE, MOLINA JM & SCADDEN DT. (1989). Haemopoietic growth factors: biology and clinical applications. *N. Engl. J. Med.*, **321**, 1449-1459.

HENDERSON IC, HAYES DF AND GELMAN R. (1988). Dose-response in the treatment of breast cancer: a critical review. *J. Clin. Oncol.*, **6**, 1501-1515.

HRYNIUK W AND BUSH H. (1984). The importance of dose intensity in chemotherapy of metastatic breast cancer. *J. Clin. Oncol.*, **2**, 1281-1288.

LICHTMAN SM, RATAIN MJ, VAN ECHO DA, ROSNER G, EGORIN MJ, BUDMAN DR, VOGELZANG NJ, NORTON L AND SCHILSKY RL. (1993). Phase I trial of granulocyte-macrophage colony-stimulating factor plus high-dose cyclophosphamide given every 2 weeks: a Cancer and Leukaemia Group B study. *J. Natl Cancer Inst.*, **85**, 1319-1326.

OSBORNE CK, SUNDERLAND CK, NEIDHART JA, RAVDIN PM AND ABELOFF MD. (1994). Failure of GM-CSF to permit dose-escalation in an every other week dose-intensive regimen for advanced breast cancer. *Ann. Oncol.*, **5**, 43-47.

VAN HOEF MEHM, BAUMANN I, LANGE C, LUFT T, DE WYNTER EA, RANSOM M, MORGENSTERN GR, YVERS A, DEXTER TM, TESTA NG AND HOWELL A. (1994). Dose-escalating induction chemotherapy supported by lenograstim preceding high-dose consolidation chemotherapy for advanced breast cancer. *Ann. Oncol.*, **5**, 217-224.

WOOD WC, BUDMAN DR, KORZUN AH, COOPER R, YOUNGER J, HART RD, MOORE A, ELLERTON JA, NORTON L, FERREE CR, BALLOW AC, FREI E AND HENDERSON IC. (1994). Dose and dose intensity of adjuvant chemotherapy for stage II, node positive breast carcinoma. *N. Eng. J. Med.*, **330**, 1253-1259.